# Retraction Note: Dapagliflozin Guards Against Cadmium-Induced Cardiotoxicity via Modulation of IL6/STAT3 and TLR2/TNFα Signaling Pathways

**DOI:** 10.1007/s12012-023-09787-5

**Published:** 2023-03-01

**Authors:** Marwa M. M. Refaie, Rehab Ahmed Rifaai, Michael Atef Fawzy, Sayed Shehata

**Affiliations:** 1 Department of Pharmacology, Faculty of Medicine, Minia University, El-Minia, 61511 Egypt; 2 Department of Histology and Cell Biology, Faculty of Medicine, Minia University, El-Minia, 61511 Egypt; 3 Department of Biochemistry, Faculty of Pharmacy, Minia University, El-Minia, 61511 Egypt; 4 Department of Cardiology, Faculty of Medicine, Minia University, El-Minia, 61511 Egypt

**Retraction Note: Cardiovascular Toxicology (2022) 22:916–928** **https://doi.org/10.1007/s12012-022-09768-0**

The Editor in Chief has retracted this article because of the following issues:

- I﻿t appears a GADPH has been re-used in Figs. 1A, 1B and 2B.
- In Fig. 1, TNFa and NFkB look very similar to each other.
- In Fig. 2B, the first and last lanes of the GPx blot look very similar to each other.

The authors provided raw data which did not contradict these concerns and stated they did use the same GAPDH blot in the 3 figures. The Editor in Chief, therefore, has lost confidence in the integrity of the article's findings. None of the authors agree to this retraction.

